# Experiences of the first two pandemic years (2020 and 2021) in regards of the alteration of violent suicide attempts compared to 2016-2021. Presenting demographic features. Research made at Dr. Manninger Jenő National Traumatology Center, Hungary

**DOI:** 10.1192/j.eurpsy.2024.1643

**Published:** 2024-08-27

**Authors:** N. M. Szeifert, B. Sebők, S. Szilágyi, M. Bérdi

**Affiliations:** ^1^Doctoral School of Psychology; ^2^Clinical Psychology and Addictology, ELTE Eötvös Lóránd University; ^3^Psychotherapy, National Institute of Sports Medicine; ^4^School of PhD Studies Workgroup for Science Management, Semmelweis University; ^5^Psychiatry and Crisis Intervention, Péterfy Hospital, Budapest, Hungary

## Abstract

**Introduction:**

During the pandemic years in Hungary the completed suicide rates hasrisen significantly. Suicide rates had been decreasing until 2019 since 1986. In 2019, 1550 people dead by completed suicide, in 2020 this number increased to 1705, in 2021,1561 cases were registered. Violent suicide attempts represent the majority of completed suicides.

**Objectives:**

In our study we were analysing the number of alteration of violent suicide attempts between 2016-2021, focusing on the trend in the first two years of the pandemic outbreak. 228 inpatients (65,4% male, 34,6% female) gone under medical treatment due to violent suicide attempts between 2016-2021 at Dr. Manninger Jenő National Traumatology Center, Budapest, Hungary.

**Methods:**

We used an interrupted time-series analysis with Prais-Winsten regression, controlling autoagressive and seasonal effects, to estimate the effect of the pandemic years on the violent suicide attempt rates in our sample. Demographic features, risk factors for suicidal behaviours, motivation and methods were analysed by Chi-square test and cross tabulation.

**Results:**

Comparing to the previous years, in the first two pandemic years significantly has risen the number of inpatients treated because of violent suicide attempts. After the rapid change in 2020, decreasing numbers could be observed in 2021.

**Conclusions:**

Analyzing the numbers of violent suicide attempts between 2016 and 2021, an increase inthe number of attempts was observed during the first two pandemic years. Detailed demographic data and potential risk factors are also to be presented in the lecture.

**Disclosure of Interest:**

None Declared

